# Supplementary material for: Using the DNA language model, GROVER, to parse effects of sequence, chromatin and regulatory features on genome stability
Source: Bioinformatics. 2026 Aug 11;42(8):btag602. doi: 10.1093/bioinformatics/btag602 (PMC13503036; doi:10.1093/bioinformatics/btag602)
Supplement: Supplementary file 1 [file btag602_supplementary_data.zip › AdditionalFile1.pdf]

Figure S1

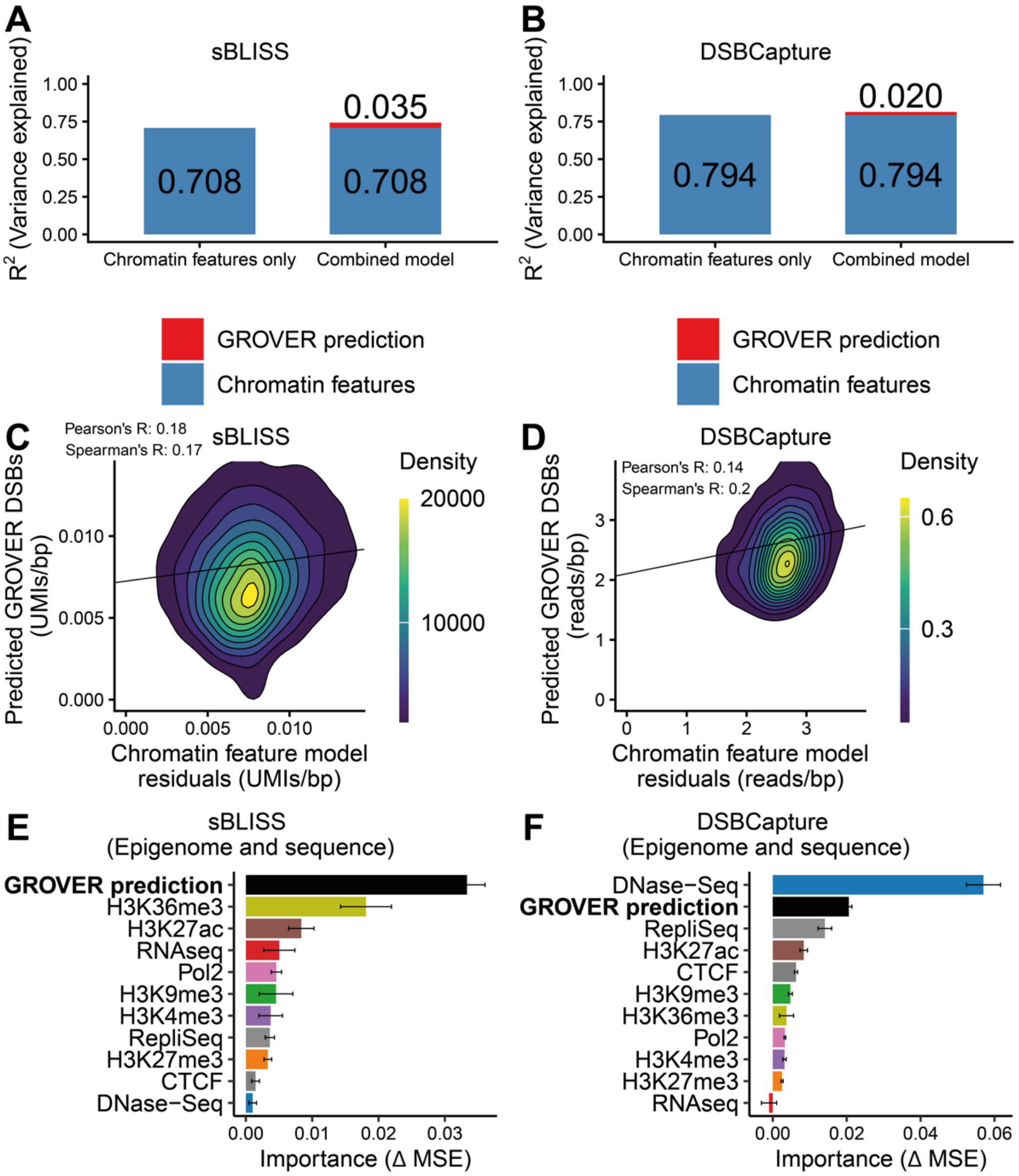

Fig. S1. Validation of GROVER importance in combine double strand break prediction model. A and B. Variance explained by chromatin features and GROVER predictions in the combined double strand break prediction model for sBLISS data (**A**) and DSBCapture data (**B**). **C and D.** Correlation between chromatin feature model residuals and GROVER predictions for sBLISS data (**C**) and DSBCapture data (**D**). **E and F.** Feature ablation importances for combined double strand break prediction model for sBLISS data (**E**) and DSBCapture data (**F**). Importances are measured using the change

in mean squared error (MSE) of the model when a feature is removed from the data and model. DSB data is shown as molecular identifier (UMI) counts (sBLISS) or read counts (DSBCapture) averaged per base pair over the length of each window.

Figure S2

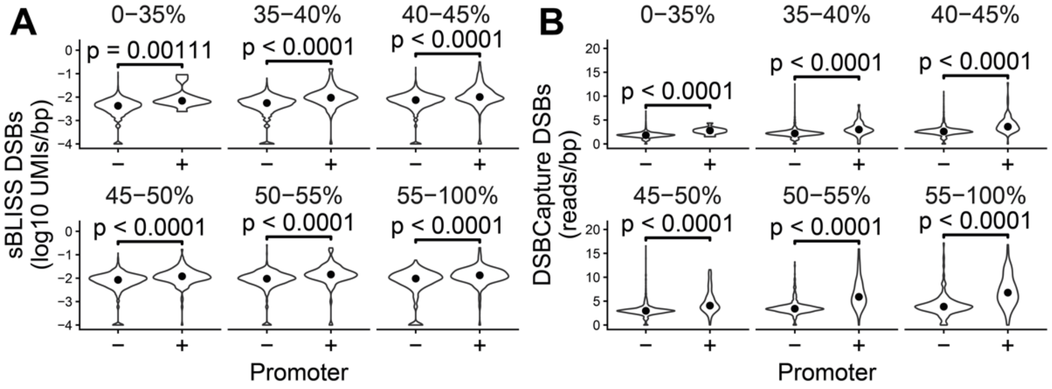

Fig. S2. Double strand break counts for promoters, stratified by GC content. A and B. Double strand break counts for promoters according to sBLISS data (**A**) and DSBCapture data (**B**).

Figure S3

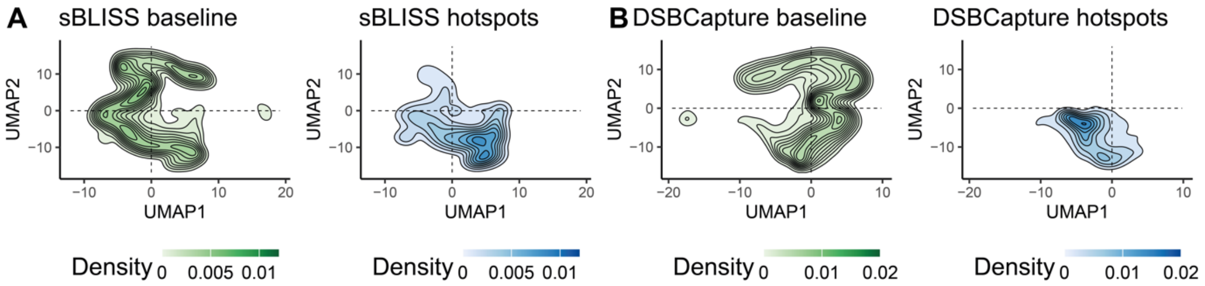

Fig. S3. Uniform manifold approximation and projection (UMAP) analysis of chromatin features. A and B. UMAP of MCF7 (**A**) and NHEK (**B**) chromatin features. Sequences are separated based on whether they are in the top 10% (hotspots) or bottom 90% (baseline) of sequences.

Figure S4

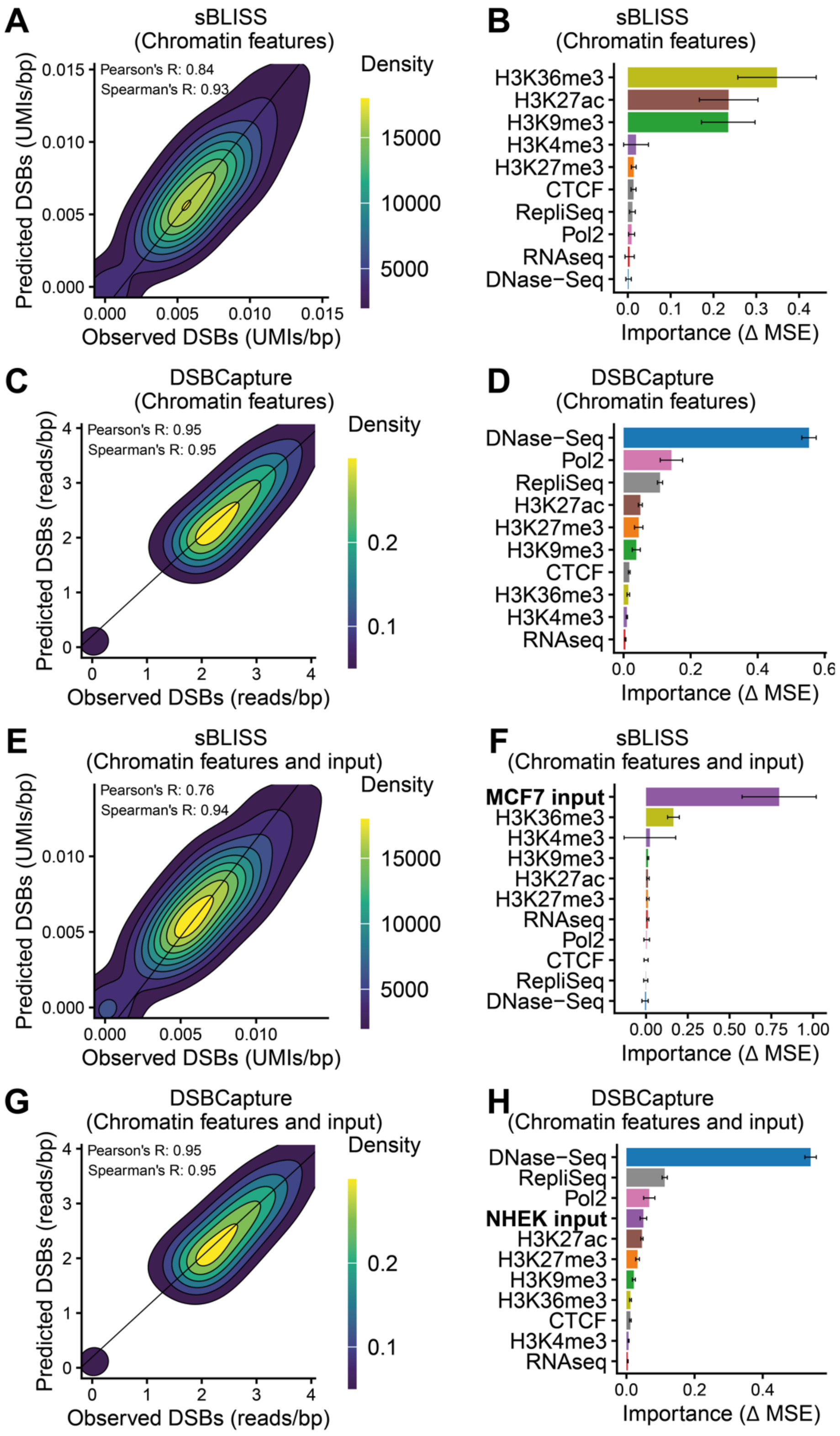

Fig. S4. Performance and importances of models that predict double strand breaks (DSBs) within 50kbp genomic windows. A and C. Performance of random forest

model trained on chromatin features to predict sBLISS data (**A**) and DSBCapture data (**C**). **B and D.** Importances of features used by the random forest model trained on chromatin features to predict sBLISS data (**B**) and DSBCapture data (**D**). **E and G.** Performance of random forest model trained on chromatin features and ChIP-Seq input data to predict sBLISS data (**E**) and DSBCapture data (**G**). **F and H.** Importances of features used by the random forest model trained on chromatin features and ChIP-Seq input data to predict sBLISS data (**F**) and DSBCapture data (**H**). DSB data is shown as molecular identifier (UMI) counts (sBLISS) or read counts (DSBCapture) averaged per base pair over the length of each window. Error bars represent standard deviation from the mean. Importances are measured using the change in mean squared error (MSE) of the model when a feature is shuffled in the test data.

Figure S5

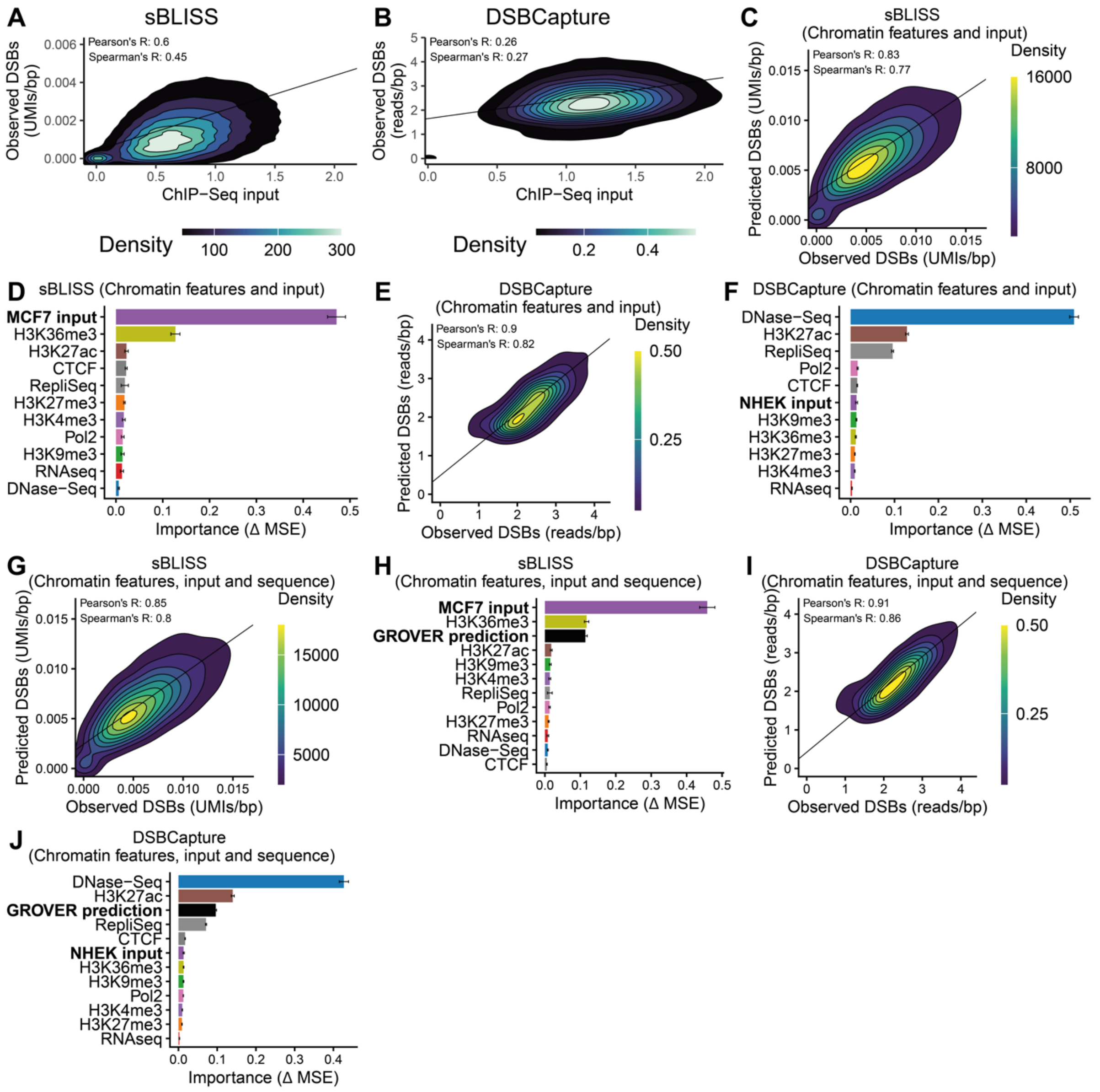

Fig. S5. Performance and importances of models including ChIP-Seq input, trained to predict double strand breaks (DSBs). A and B. Correlation between ChIP-Seq input data and observed sBLISS data (**A**) or DSBCapture data (**B**). **C and E.** Performance of the random forest model trained on chromatin features and ChIP-Seq input data to predict sBLISS data (**C**) and DSBCapture data (**E**). **D and F.** Importances of features used by the random forest model trained on chromatin features and ChIP-Seq input data to predict sBLISS data (**D**) and DSBCapture data (**F**). **G and I.** Performance of the random forest

model trained on chromatin features, ChIP-Seq input data and fine-tuned GROVER model predictions to predict sBLISS data (**G**) and DSBCapture data (**I**). **H and J.** Importances of features used by the random forest model trained on chromatin features, ChIP-Seq input data and fine-tuned GROVER model predictions to predict sBLISS data (**H**) and DSBCapture data (**J**). DSB data is shown as molecular identifier (UMI) counts (sBLISS) or read counts (DSBCapture) averaged per base pair over the length of each window. Error bars represent standard deviation from the mean. Importances are measured using the change in mean squared error (MSE) of the model when a feature is shuffled in the test data.

Figure S6 XGBoost

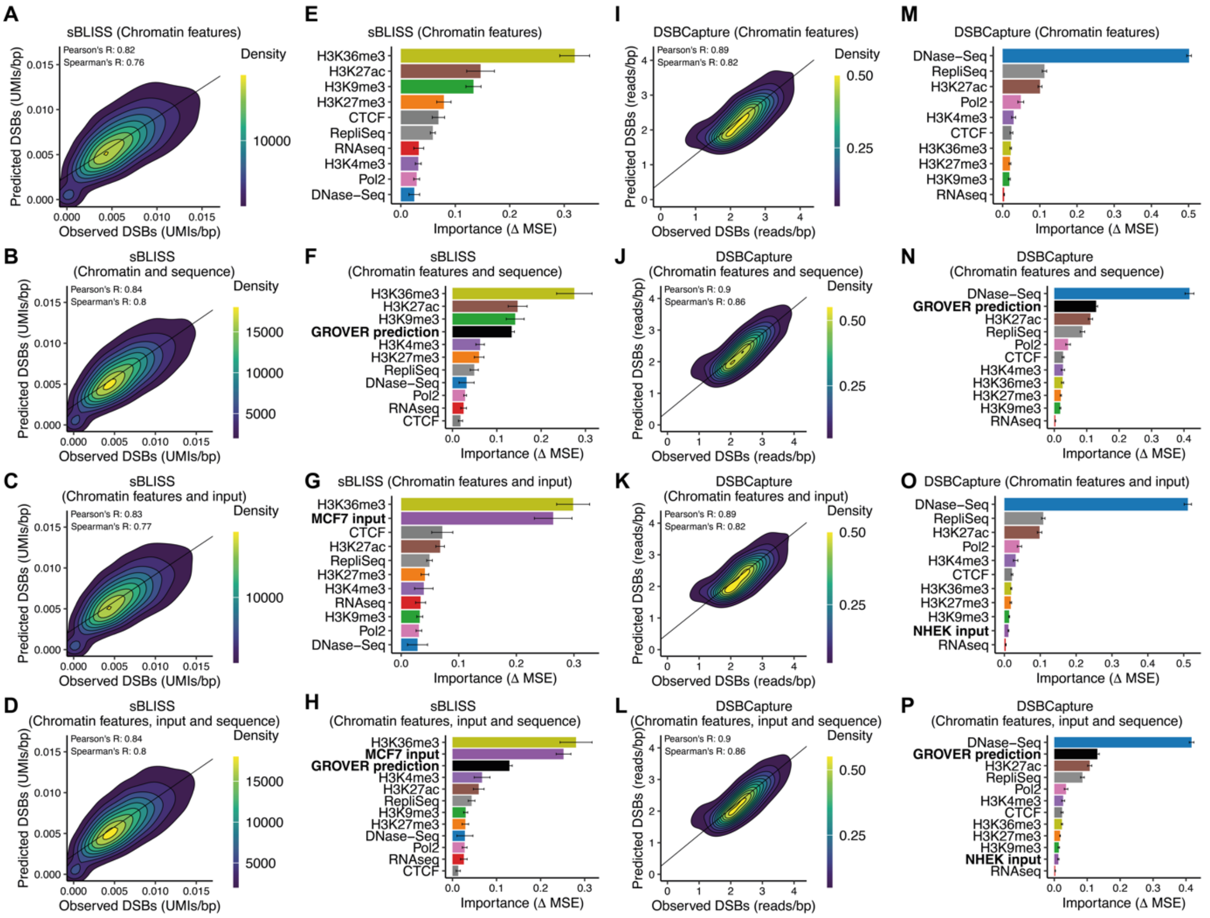

Fig. S6. Performance and importances of models using eXtreme Gradient Boosting (XGBoost). A. Performance of XGBoost model to predict sBLISS data in MCF7 cells based on genome biological features, (**B)** genome biological features and GROVER, (**C)** genome biological features and ChIP-Seq input, and (**D)** genome biological features and ChIP-Seq input and GROVER. **E.** Feature contribution of XGBoost model to predict sBLISS data based on genome biological features, (**F**) genome biological features and GROVER, (**G**) genome biological features and ChIP-Seq input, and (**H**) genome biological features and ChIP-Seq input and GROVER. **I.** Performance of XGBoost model to predict DSBCapture data in NHEK cells based on genome biological features, **(J)** genome biological features and GROVER, **(K)** genome biological features and ChIP-Seq input, and **(L)** genome biological features and ChIP-Seq input and GROVER. **M.** Feature

contribution of XGBoost model to predict sBLISS data based on genome biological features, **(N)** genome biological features and GROVER, **(O)** genome biological features and ChIP-Seq input, and **(P)** genome biological features and ChIP-Seq input and GROVER.

Figure S7 Multi Layer Perceptron (MLP)

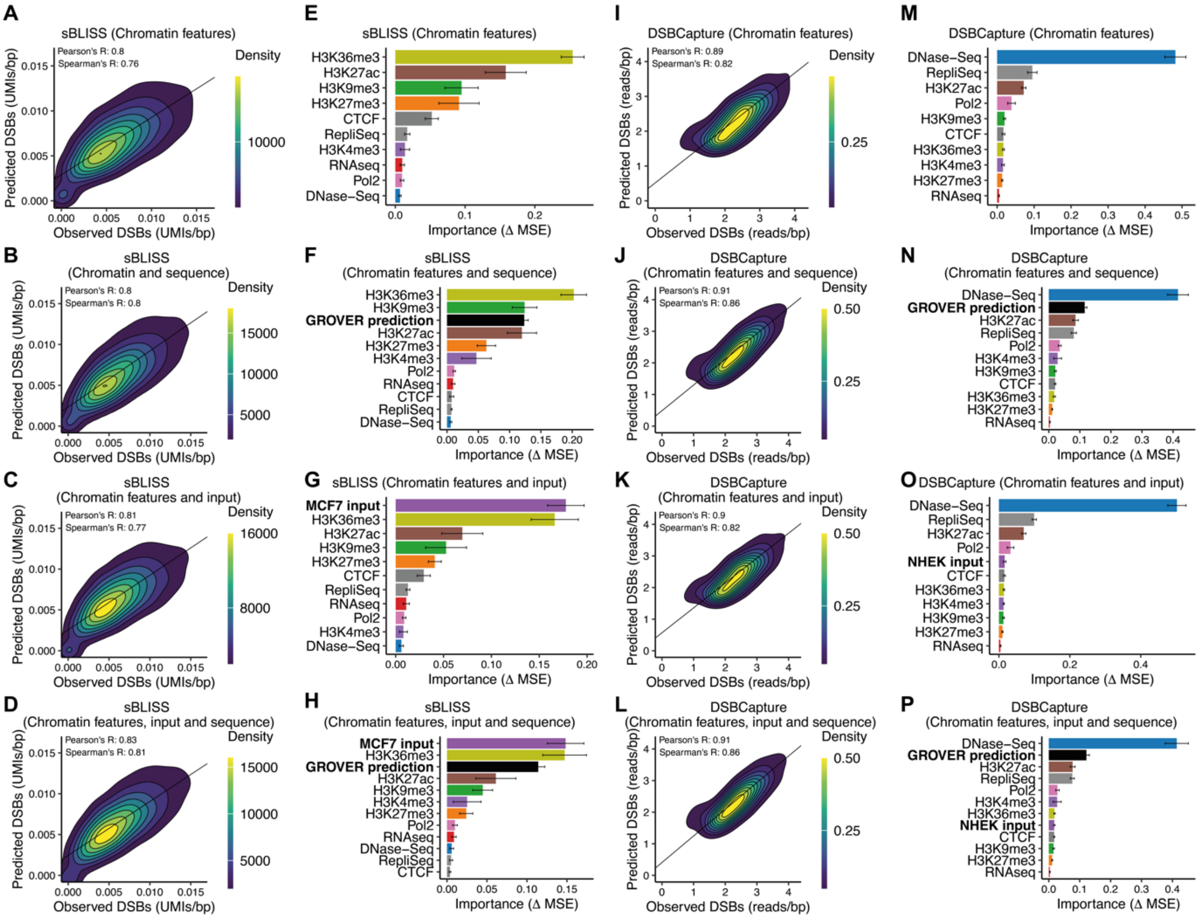

Fig. S7. Performance and importances of models using Multilayer Perceptrons (MLP). A. Performance of MLP model to predict sBLISS data in MCF7 cells based on genome biological features, (**B)** genome biological features and GROVER, (**C)** genome biological features and ChIP-Seq input, and (**D)** genome biological features and ChIP-Seq input and GROVER. **E.** Feature contribution of XGBoost model to predict sBLISS data based on genome biological features, (**F**) genome biological features and GROVER, (**G**) genome biological features and ChIP-Seq input, and (**H**) genome biological features and ChIP-Seq input and GROVER. **I.** Performance of MLP model to predict DSBCapture data in NHEK cells based on genome biological features, **(J)** genome biological features and GROVER, **(K)** genome biological features and ChIP-Seq input, and **(L)** genome biological features and ChIP-Seq input and GROVER. **M.** Feature contribution of

XGBoost model to predict sBLISS data based on genome biological features, **(N)** genome biological features and GROVER, **(O)** genome biological features and ChIP-Seq input, and **(P)** genome biological features and ChIP-Seq input and GROVER.

Figure S8 Light GBM

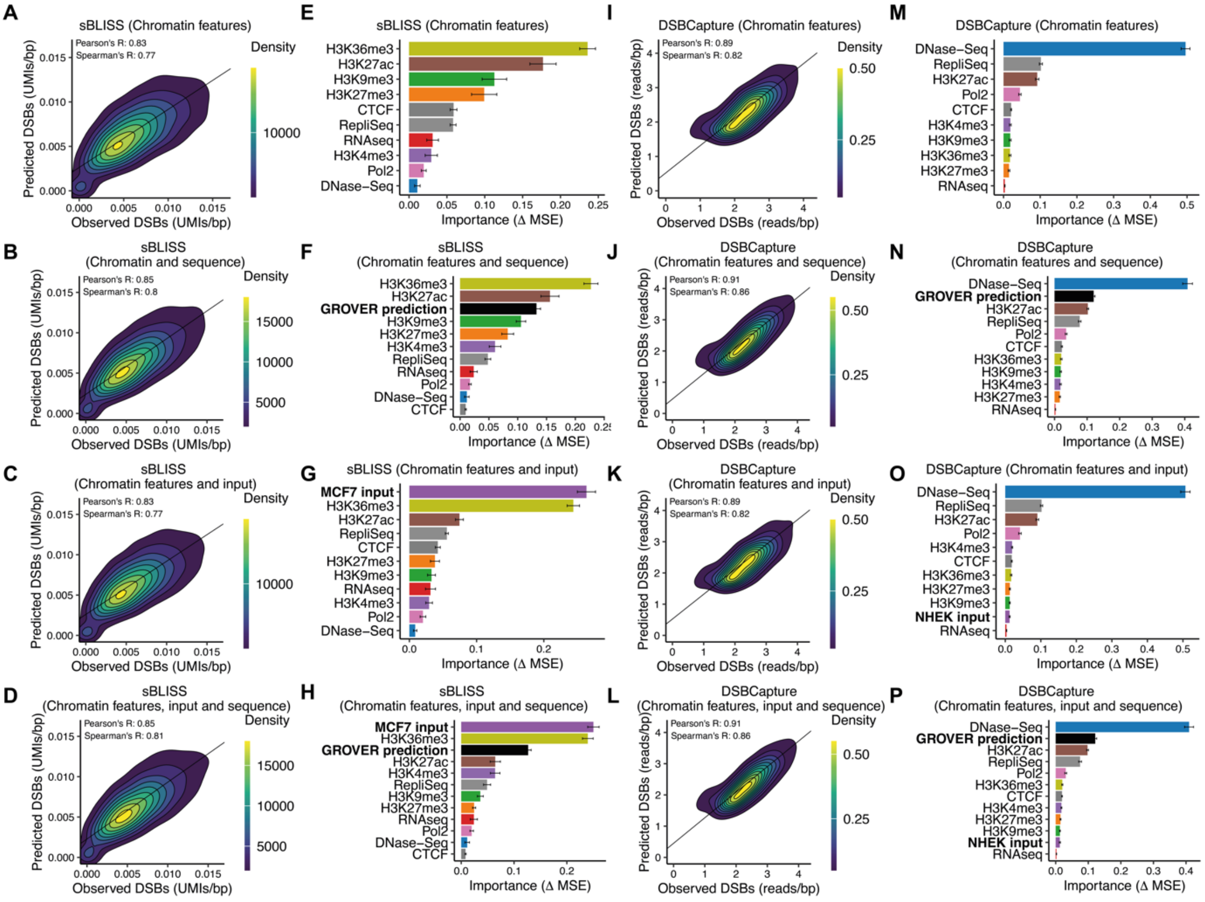

Fig. S8. Performance and importances of models using Light Gradient Boosting Machine (LightGBM). A. Performance of LightGBM model to predict sBLISS data in MCF7 cells based on genome biological features, **(B)** genome biological features and GROVER, **(C)** genome biological features and ChIP-Seq input, and **(D)** genome biological features and ChIP-Seq input and GROVER. **E.** Feature contribution of XGBoost model to predict sBLISS data based on genome biological features, (**F**) genome biological features and GROVER, (**G**) genome biological features and ChIP-Seq input, and (**H**) genome biological features and ChIP-Seq input and GROVER. **I.** Performance of LightGBM model to predict DSBCapture data in NHEK cells based on genome biological features, **(J)** genome biological features and GROVER, **(K)** genome biological features and ChIP-Seq input, and **(L)** genome biological features and ChIP-

Seq input and GROVER. **M.** Feature contribution of XGBoost model to predict sBLISS data based on genome biological features, **(N)** genome biological features and GROVER, **(O)** genome biological features and ChIP-Seq input, and **(P)** genome biological features and ChIP-Seq input and GROVER.

Figure S9 Linear Regression

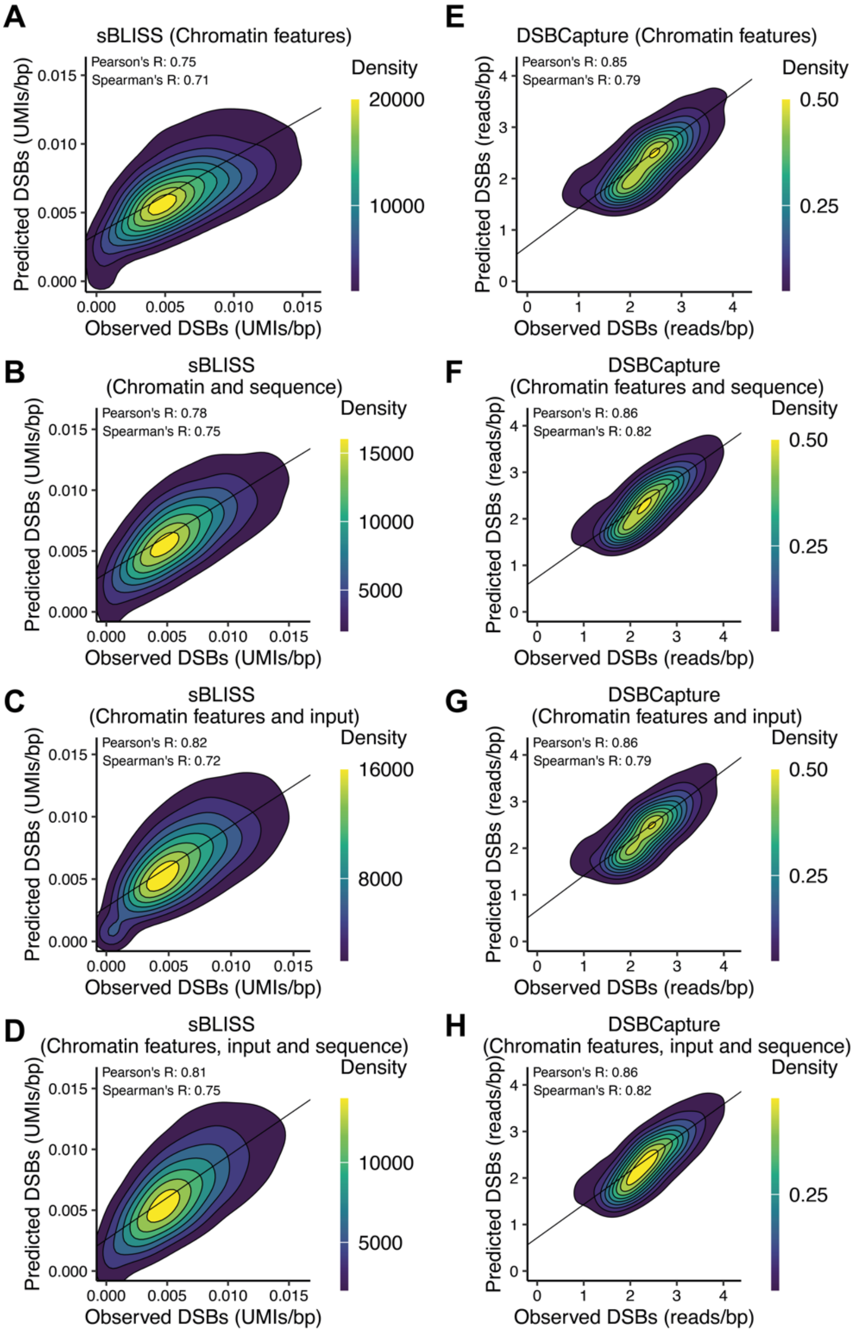

Fig. S9. Performance of models using Linear Regression. A. Performance of a Linear Regression model to predict sBLISS data in MCF7 cells based on genome biological features, (**B)** genome biological features and GROVER, (**C)** genome biological features and ChIP-Seq input, and (**D)** genome biological features and ChIP-Seq input and GROVER.

Figure S10

A

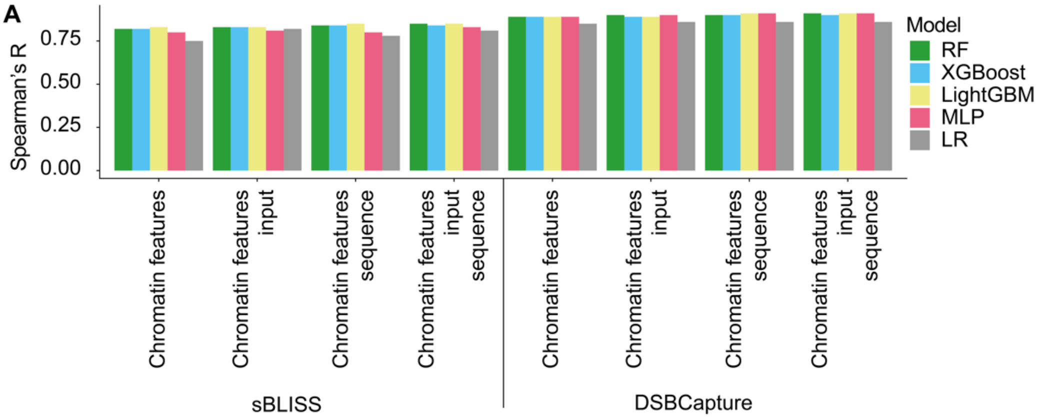

B

		Spearman's R				
		RF	XGBoost	LightGBM	MLP	LR
sBLISS	Chromatin features	0.76	0.76	0.77	0.76	0.71
	Chromatin features and sequence	0.8	0.8	0.8	0.8	0.75
	Chromatin features and input	0.77	0.77	0.77	0.77	0.72
	Chromatin features, input and sequence	0.8	0.8	0.81	0.81	0.75
DSBCapture	Chromatin features	0.82	0.82	0.82	0.82	0.79
	Chromatin features and sequence	0.86	0.86	0.86	0.86	0.82
	Chromatin features and input	0.82	0.82	0.82	0.82	0.79
	Chromatin features, input and sequence	0.86	0.86	0.86	0.86	0.82

C

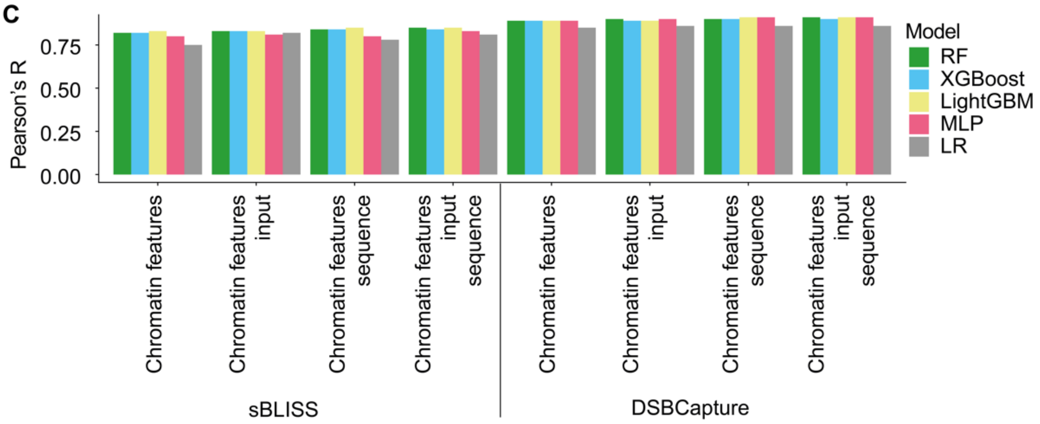

D

		Pearson's R				
		RF	XGBoost	LightGBM	MLP	LR
sBLISS	Chromatin features	0.82	0.82	0.83	0.8	0.75
	Chromatin features and sequence	0.84	0.84	0.85	0.8	0.78
	Chromatin features and input	0.83	0.83	0.83	0.81	0.82
	Chromatin features, input and sequence	0.85	0.84	0.85	0.83	0.81
DSBCapture	Chromatin features	0.89	0.89	0.89	0.89	0.85
	Chromatin features and sequence	0.9	0.9	0.91	0.91	0.86
	Chromatin features and input	0.9	0.89	0.89	0.9	0.86
	Chromatin features, input and sequence	0.91	0.9	0.91	0.91	0.86

Fig. S10. Performance benchmarking. A & B. Spearman's R for Random Forest (RF), eXtreme Gradient Boosting (XGBoost), Light Gradient Boosting Machine (LightGBM), Multy Layer Perceptron (MLP), and Linear Regression (LR) for different sets of input data to predict sBLISS in MCF7 cells and DSBCapture in NHEK cells. **C & D.** Pearson's R for Random Forest (RF), eXtreme Gradient Boosting (XGBoost), Light Gradient Boosting

Machine (LightGBM), Multy Layer Perceptron (MLP), and Linear Regression (LR) for different sets of input data to predict sBLISS in MCF7 cells and DSBCapture in NHEK cells.

Figure S11

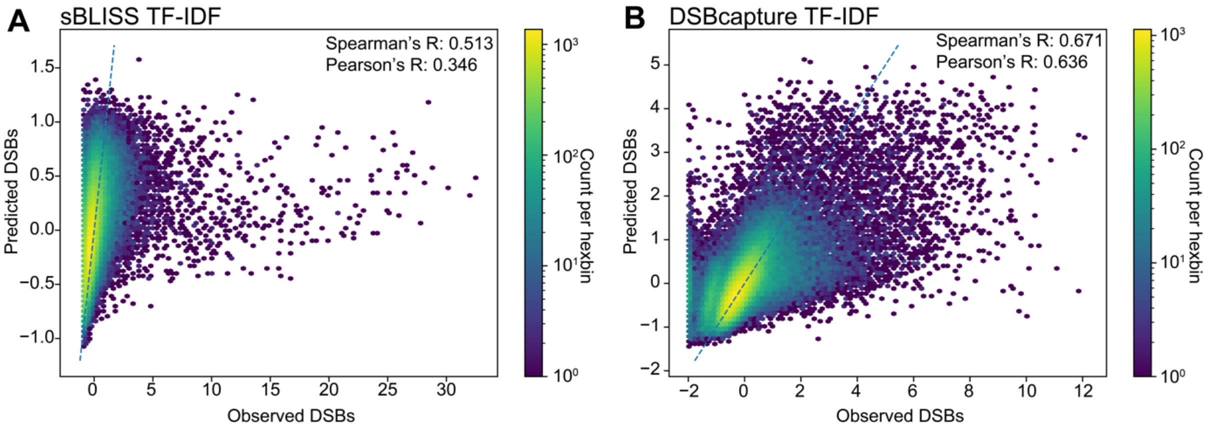

Fig. S11. Term-Frequency-Inverse Document Frequency (TF-IDF) sets a base-line prediction for dependencies of DSB locations on hexamer distribution in the human genome. A. A ridge regression model to predict sBLISS data in MCF7 cells using TF-IDF. **B.** A ridge regression model to predict DSBCapture data in NHEK cells using TF-IDF.
